# Pre-treatment direct costs for people with tuberculosis during the COVID-19 pandemic in different healthcare settings in Bandung, Indonesia

**DOI:** 10.1371/journal.pone.0320401

**Published:** 2025-04-01

**Authors:** Bony Wiem Lestari, Eka Saptiningrum, Lavanya Huria, Auliya Ramanda Fikri, Benjamin Daniels, Nathaly Aguilera Vasquez, Angelina Sassi, Jishnu Das, Charity Oga-Omenka, Susan M. McAllister, Madhukar Pai, Bachti Alisjahbana

**Affiliations:** 1 Tuberculosis Working Group, Research Center for Care and Control of Infectious Disease Universitas Padjadjaran, Bandung, Indonesia; 2 Department of Public Health, Faculty of Medicine, Universitas Padjadjaran, Bandung, Indonesia; 3 Department of Internal Medicine, Radboud Institute for Health Sciences, Radboud University Medical Center, Nijmegen, The Netherlands; 4 Department of Epidemiology, Biostatistics and Occupational Health, McGill University, Montreal, Canada; 5 McGill International TB Center, Montreal, Canada; 6 McCourt School of Public Policy, Georgetown University, Washington, District of Columbia, United States of America; 7 School of Public Health Sciences, University of Waterloo, Waterloo, Canada; 8 Centre for International Health, University of Otago, Dunedin, New Zealand; 9 Department of Internal Medicine, Faculty of Medicine Universitas Padjadjaran, Hasan Sadikin General Hospital, Bandung, Indonesia; National Research and Innovation Agency, INDONESIA

## Abstract

The tuberculosis (TB) program was massively disrupted due to the COVID-19 pandemic, which may have impacted on an increase in costs for people with TB (PWTB) and their households. We aimed to quantify the pre-treatment out-of-pocket costs and the factors associated with these costs from patients’ perspective during the COVID-19 pandemic in Bandung, Indonesia. Adults with pulmonary TB were interviewed using a structured questionnaire for this cross-sectional study recruiting from 7 hospitals, 59 private practitioners, and 10 community health centers (CHCs) between July 2021 to February 2022. Costs in rupiah were converted into US dollars and presented as a median and interquartile range (IQR). Factors associated with costs were identified using quantile regression. A total of 252 participants were recruited. The median total pre-treatment cost was $35.45 (IQR 17.69-67.62). The highest median cost was experienced by participants from private hospitals ($54.51, IQR 29.48-98.47). The rapid antigen and PCR for SARS-CoV-2 emerged as additional medical costs among 26% of participants recruited in private hospitals. Visiting ≥  6 providers before diagnosis ($38.40 versus $26.20, p <  0.001), presenting first at a private hospital ($50.68, p <  0.05) and private practitioners ($34.97, p <  0.05), and being diagnosed in the private health sector ($39.98 versus $20.30, p <  0.05) were significantly associated with higher pre-treatment costs. PWTB experienced substantial out-of-pocket costs in the process of diagnosis during the COVID-19 pandemic despite free TB diagnosis and treatment. Early detection and identification play an important role in reducing pre-diagnostic TB costs.

## Introduction

Tuberculosis (TB) is one of the leading causes of infectious disease burden globally. In 2021, an estimated 10.6 million people fell ill with TB, yet only 6.4 million new cases were officially diagnosed, marking a significant decrease from 2019, largely attributed to the impact of the Coronavirus Disease 2019 (COVID-19) pandemic. Moreover, there were 1.4 million reported TB-related deaths in 2021 [1]. TB often places a large financial burden on People with TB (PWTB) and their families due to costs incurred from medical care and loss of income. In 2022, the World Health Organization (WHO) reported that 48% of PWTB were dealing with catastrophic costs [1] - defined as total direct and indirect costs 20% above the annual household income during a TB episode [2]. This was reiterated in a systematic review and meta-analysis in 2022 which reported 43% of households affected by TB faced catastrophic costs [3]. The WHO target for zero percent of individuals with TB facing catastrophic costs was not reached by 2020, causing the deadline to be moved to 2025 [1,4]. The financial burden incurred by PWTB remains an obstacle to optimal TB management globally [3], including timely diagnosis and successful completion of treatment [5].

Indonesia has the second largest burden of TB cases in the world [1]. In 2022, there were 694,808 TB cases diagnosed [6]. The WHO estimates the actual number of cases in Indonesia to be around 2.4 times the number of actual notified cases – mainly due to gaps in the notification and reporting systems, as well as underreporting of cases in the private sector [4]. Acknowledging the financial burden of TB, the Indonesian government provides TB services for free under its National TB Program (NTP), supported by the Indonesian Universal Health Coverage (UHC) scheme [7, 8].

Despite around 235.7 million individuals or ~ 85.2% of the total Indonesian population being enrolled for National Health Insurance (NHI for short, or Badan Penyelenggara Jaminan Sosial (BPJS) in Indonesian) [9], PWTB continue to incur costs while seeking care for TB, especially if they first seek care from private healthcare providers – a common occurrence in Indonesia [10]. In 2019, 26.5% of PWTB in Indonesia were reported to experience catastrophic costs [10, 11].

During the COVID-19 pandemic, there were massive disruptions to health services, including services for TB, such as closure of facilities, the rearrangements of healthcare service priorities, reduced health promotion and TB prevention programs, and limited contact tracing and TB testing [12–14]. These led to an almost 20% decrease in case notifications [1]. To help overcome this, the Ministry of Health created a TB Service Protocol to keep TB services running during the pandemic. The Circular Letter PM.01.02/1/840/2020 was issued, which regulates various aspects, such as TB precautions, management, and laboratory services [15].

Despite the presence of the TB Service Protocol, the COVID-19 pandemic-related disruptions may lead to increasing healthcare costs for PWTB and their household due to the delay in seeking appropriate TB management, thereby increasing the risk of greater severity and complications [5,16]. Moreover, a survey of 12,216 households in all 34 provinces in Indonesia and across all income groups showed 74.3% of households reporting a decline in income during the COVID-19 pandemic due to the restrictions imposed by local and national government [17]. This income decline and the potential increase in TB-related costs had the combined potential to increase the number of households facing catastrophic costs [16].

Understanding the financial burden of TB is crucial to provide evidence to determine appropriate interventions by the national government and relevant stakeholders, and to advocate for adequate financing for programs and interventions. The COVID-19 pandemic-related economic issues further necessitated the need to understand this matter in the context of the pandemic. Therefore, this study aimed to describe direct pre-treatment costs for PWTB between public and private healthcare providers, as well as the factors associated with these costs during the COVID-19 pandemic.

## Materials and methods

### Study design and setting

This cross-sectional study followed a similar design and setting to a previous study conducted by our research team in 2017, which recruited PWTB prior to the COVID-19 pandemic in Bandung, Indonesia [10] and provided a pre-COVID description of patient costs. Our study was a part of another study regarding COVID-19 Effect of Tuberculosis or COVET project [18], which discussed the impact of the COVID-19 pandemic on the private health sector and tuberculosis services in three countries that was carried out in Bandung. Bandung is the third-largest metropolitan area in Indonesia with a population of more than 2.5 million people. In 2021, more than eight thousand TB cases were registered and treated in the city [19, 20].

### Sample size, sampling, and eligibility criteria

Participants were recruited from 36 (of 80) randomly selected Community Health Center (CHC) areas in Bandung municipality [21]. Participants eligible were adults (aged ≥  18 years), newly diagnosed drug-sensitive TB or having been on pulmonary TB treatment, were within their first two months of treatment, and a resident in Bandung City for a minimum of 6 months. People with a history of anti-TB treatment in the past 6 months or having communication problems (such as having a speech impediment and intellectual disability) were excluded.

Within the 36 CHC areas, 10 CHCs, 4 (of 12) private hospitals, and 3 (of 7) public hospitals were sampled purposively by examining the patient list and selecting the providers which had the greatest number of PWTB fitting the inclusion criteria.

The willingness of and permission from the health providers to participate were also factors impacting on the inclusion of recruitment sites. A total of 72 (of 273) private practitioners also reported diagnosing and/or treating PWTB during the study period, of whom 59 were willing to participate during the data collection period. This study was conducted as part of the COVET project, where the sample sizes were determined in advance due to funding and time constraints. Data collection began in private facilities in July 2021 and in public sector facilities in October 2021, continuing until February 2022. Patients who gave their consent were recruited until the target sample size was achieved. Aiming for a power of 80% at the 5% alpha level the sample size was calculated to be 197 and adjusting for a non-response rate of 15% then rounded up to 250.

### Data collection

Information on socio-demographic characteristics, clinical history, healthcare seeking pathway, and pre-treatment cost information was collected using a structured questionnaire form, using the “Tool to Estimate Patient Costs” following the corridor of WHO Tuberculosis Patient Cost Survey (TBPCS) methodology [2,22] which had been adapted for the Indonesian setting [23], along with some additional questions regarding the COVID-19 pandemic. The questionnaire was translated to, and conducted in, Bahasa Indonesia, the official and national language of Indonesia and pre-piloted with six individuals. Informed consent was obtained before the interview which was conducted by trained research assistants through in-person (performed according to the local COVID-19 regulations) or online calls. Participants were provided transportation money or internet credit directly transferred into their account, or as cash, totalling US$6-$10, depending on the interview method used as appreciation for their involvement. Data obtained from the participants were documented on paper-based questionnaires, and entered to REDCap version 12.14.12 (data management platform) immediately after the interview [24,25]. The data was validated by a research assistant to ensure quality and completeness.

### Definitions

- Direct cost is the amount of money spent on medical and non-medical costs throughout the care seeking process, net of any reimbursements.
- Medical direct cost is the sum of any expenses incurred related to medical costs by PWTB or persons who accompanied them, minus any reimbursements, while non-medical direct costs are the sum of transportation, food, supplements, etc [2].
- Hospitalization costs are any costs paid during the participant’s stay in hospital, including costs for anyone who accompanied them during their hospital stay.
- COVID-19 screening cost is the expenditure related specifically to rapid antigen test and/or Polymerase Chain Reaction (PCR) test in this study.
- Informal providers refer to individuals or entities that offer healthcare services or products outside the formal healthcare system, such a drug store, pharmacy, etc.
- Financial standing was measured by two indicators: adequacy of household income, and status of financial management – defined as adequate, barely adequate, and inadequate. Financial standing was defined as adequate if people with TB and their families have no difficulties to provide for their needs every month.
- All data for this study were related to costs before and up to the time of TB diagnosis (prior to treatment initiation).

### Data analysis

After the desired sample size was met and all interviews were concluded, the data was exported to SPSS version 26 for analysis. Cost-related responses that were recorded in Indonesian Rupiah (IDR) were converted into United States Dollar (USD) using the World Bank 2021 exchange rate (14.582 IDR is 1 USD) [26].

Descriptive statistics reported included frequency, percentage, median, and interquartile range stratified by recruitment site. Quantile regression analysis, to compare medians, was used to investigate associations between participants’ characteristics with the pre-treatment direct cost. We included age and gender, both identified as confounders from previous literature, and other variables that had a p-value of < 0.2 in the univariable analysis to the adjusted multivariable quantile regression analysis. Coefficients and 95% confidence intervals are reported to indicate the difference in medians between groups.

### Ethics statement

The Institutional Review Boards (IRB) at Universitas Padjadjaran (166/UN6.KEP/EC/2021), McGill University (IRB Internal Study Number: A04-M43-22A), and Research Institute of McGill University Health Center (Covid BMGF/ 2021-7197) approved the study protocol. Before the interview, we provided all respondents with a written and oral explanation of the study. Only those who had signed an informed consent form were interviewed.

## Results

### Characteristics of the participants

**Fig 1:**
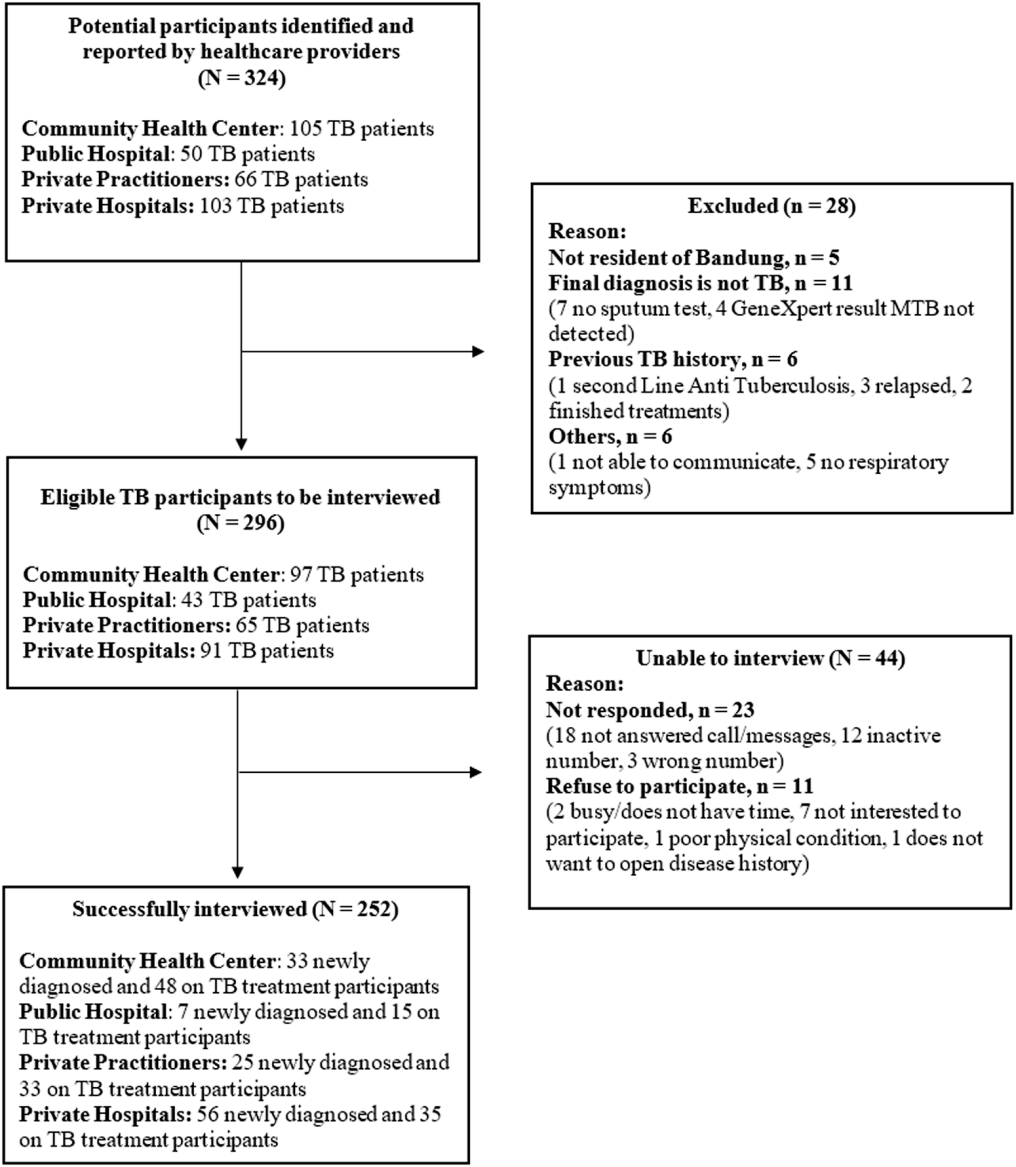
Flowchart of study participants (N ** = 252)**. First care-seeking visit to an informal provider upon becoming unwell was reported by half of the participants (50.0%; n = 126). More than half (56.7%; n = 143) visited 6 or more healthcare providers before they were diagnosed with TB – and this proportion was considerably higher in PWTB recruited in private practice (77.6%; n = 45). For over a third of participants (37.7%; n = 95), it was more than 60 days after experiencing symptoms that they visited a formal health care provider, and for over a half (55.6%; n = 140), it took more than 14 days from the first formal or non-formal health care provider visits to get a diagnosis. Around half of the participants (55.2%; n = 139) had a total duration of ≥  60 days from the onset of symptoms to diagnosis. Being hospitalized before being diagnosed with TB was reported by 42 (16.7%) participants, all of whom were recruited from hospitals.

From participating healthcare facilities in the study area, 324 PWTB were identified of whom 28 (8.6%) were ineligible, leaving 296 eligible participants and 44 (14.9% of 296) were unable to be interviewed, giving 252 (85.1% of 296) who participated (Fig 1). 82.5% (208/252) of the participating patients were interviewed within 60 days after their TB diagnosis. Among the 252 PWTB who were interviewed, 41 participants (16.3% of 252) had a history of TB-related hospitalization. The participants’ characteristics are described in Table 1. The overall median (IQR) age was 36 (25-58) years (which was greater in PWTB recruited in public and private hospitals compared with CHCs and private practice) and 132 (52.4%) were male. Just over a half of the participants were married (54.0%; n = 136), and 56.7% (n = 143) lived in households of ≤ 4 people. Almost two-thirds (63.5%; n = 160) had an education of high school completion or more. Comorbidity was self-reported by 67 (26.7%) participants. These conditions included Human Immunodeficiency Virus or HIV (n =  3), diabetes (n =  34), hypertension (n =  18), dyslipidemia (n =  5), heart disease (n =  6), asthma (n =  19). Participants might report more than one comorbidity. The majority of participants had health insurance (84.1%; n = 211) – which was slightly lower in those recruited in CHCs (76.5%; n = 62). Half (51.2%; n = 129) had an income earning job, but only 95 (37.7%) were the primary income earner in their household. Almost half (47.2%; n = 119) did not report their household income and of those who did (n = 133), 66.9% had a household income less than the minimum wage of $256.6 per month.

**Table 1:** Sociodemographic, clinical, and health seeking characteristics of study participants according to recruitment site (N =  252).

Characteristic, n (%)	Community Health Center n = 81 (32%)	Private practitioner n = 58 (23%)	Public hospital n = 22 (9%)	Private hospital n = 91 (36%)	Total N = 252 (100%)
**Gender**					
Male	37 (45.7)	30 (51.7)	16 (72.7)	49 (53.8)	132 (52.4)
Female	44 (54.3)	28 (48.3)	6 (27.3)	42 (46.2)	120 (47.6)
**Age, median (IQR)**	29 (21-43)	33 (24-54)	50 (35-55)	42 (26-61)	36 (23-58)
**Age group**					
18-29	42 (51.9)	27 (46.6)	5 (22.7)	29 (31.9)	103 (40.9)
30-39	15 (18.5)	8 (13.8)	2 (9.1)	14 (15.4)	39 (15.5)
40-49	12 (14.8)	5 (8.6)	3 (13.6)	12 (13.2)	32 (12.7)
50^+^	12 (14.8)	18 (31)	12 (54.5)	36 (39.6)	78 (31.0)
**Marital status**					
Married	35 (43.2)	35 (60.3)	13 (59.1)	53 (58.2)	136 (54.0)
Single/divorced/widowed	46 (56.8)	23 (39.7)	9 (40.9)	38 (41.8)	116 (46.0)
**Comorbidities (self-reported)**					
Yes	13 (16.0)	16 (27.6)	6 (27.3)	32 (35.2)	67 (26.7)
**Education**					
Primary completed or less	12 (14.8)	9 (15.5)	10 (45.5)	19 (20.9)	50 (19.8)
Secondary completed	14 (17.3)	13 (22.4)	1 (4.5)	14 (15.4)	42 (16.7)
High school completed	46 (56.8)	29 (50)	10 (45.5)	43 (47.3)	128 (50.8)
College/university/posts-graduate	9 (11.1)	7 (1s2.1)	1 (4.5)	15 (16.5)	32 (12.7)
**Insurance**					
NHI (Government insurance)/private	62 (76.5)	47 (81)	22 (100)	81 (89.0)	211 (84.1)
**Number of people in the household**					
≤ 4	46 (56.8)	33 (56.9)	11 (50)	53 (58.2)	143 (56.7)
> 4	35 (43.2)	25 (43.1)	11 (50)	38 (41.8)	109 (43.3)
**Current income earning job**					
Yes	37 (45.7)	31 (53.4)	12 (54.5)	49 (53.8)	129 (51.2)
**Household primary income earner**					
Participant	29 (35.8)	21 (36.2)	11 (50)	34 (37.4)	95 (37.7)
Other	52 (64.2)	37 (63.8)	11 (50)	57 (62.6)	157 (62.3)
**Household income** ^*^					
<$256.6	32 (39.5)	24 (41.4)	10 (45.5)	23 (25.3)	89 (35.3)
≥ $256.6	9 (11.1)	10 (17.2)	3 (13.6)	22 (24.2)	44 (17.5)
Not available	40 (49.4)	24 (41.4)	9 (40.9)	46 (50.5)	119 (47.2)
**First health care provider visited to seek treatment**					
Informal provider	44 (54.3)	31 (53.4)	7 (31.8)	44 (48.4)	126 (50.0)
Private Practitioner	23 (28.4)	24 (41.4)	11 (50)	24 (26.4)	82 (32.5)
Community Health Center	12 (14.8)	1 (1.7)	2 (9.1)	7 (7.7)	22 (8.7)
Private hospital	1 (1.2)	2 (3.4)	1 (4.5)	16 (17.6)	20 (7.9)
Public hospital	1 (1.2)	0 (0)	1 (4.5)	0 (0)	2 (0.8)
**Hospitalization history**					
Yes	0 (0)	0 (0)	11 (50)	31 (34.1)	42 (16.7)
**Number of encounters with any health care provider before TB diagnosis**					
<6 visits	38 (46.9)	13 (22.4)	13 (59.1)	45 (49.5)	109 (43.3)
≥6 visits	43 (53.1)	45 (77.6)	9 (40.9)	46 (50.5)	143 (56.7)
**Duration from symptoms to first formal health care provider visit (days)**					
1-30	35 (43.2)	28 (48.3)	12 (54.5)	32 (35.2)	107 (42.5)
31-60	18 (22.2)	11 (19.0)	4 (18.2)	17 (18.7)	50 (19.8)
>60	28 (34.6)	19 (32.8)	6 (27.3)	42 (46.2)	95 (37.7)
**Duration from first formal/non-formal health care provider visit to TB diagnosis (days)**					
1-7	27 (33.3)	18 (31.0)	6 (27.3)	30 (33.0)	81 (32.1)
8-14	14 (17.3)	7 (12.1)	2 (9.1)	8 (8,8)	31 (12.3)
>14	40 (49.4)	33 (56.9)	14 (63.6)	53 (58.2)	140 (55.6)
**Duration from the first symptoms to TB diagnosis (days)**					
< 60	37 (45.7)	25 (43.1)	9 (40.9)	42 (46.2)	113 (44.8)
≥ 60	44 (54.3)	33 (56.9)	13 (59.1)	49 (53.8)	139 (55.2)

* Income was divided by the minimum wage of Bandung City in 2021 (Rp 3.742.276 equivalent to $256.6)

### Pre-treatment direct costs

The median (IQR) sub-total of direct medical and non-medical costs incurred for 250 PWTB until diagnosis was $29.80 (IQR 15.62-55.84) with the highest cost reported by participants recruited from the private sector (Private hospitals: n = 90; $41.62, IQR 17.46-80.47: Private practitioners: n = 58; $39.07, IQR 21.79-66.50) (Table 2). Two participants spent nothing on pre-treatment cost (excluding hospitalization cost) and were not included in sub-total cost analysis. The median direct medical cost was greater than non-medical ($22.29, IQR 8.91-47.59 vs $8.22, IQR 4.80-13.68), and was again higher among participants who were recruited in the private health sector; $32.95 (IQR 14.02-52.10) and $32.06 (IQR 9.73-74.83) incurred by participants recruited through private practitioners and private hospitals, respectively. Of the direct medical costs, chest radiography ($10.97, IQR 5.49-18.99) and medication ($9.26, IQR 4.49-21.05) costs were the highest in all recruitment sites, except for recruitment through private practitioners where a small number (n = 4), incurred costs for a rapid antigen test ($15.42, IQR 9.34-17.14). Non-medical costs were highest in private hospitals ($9.33, IQR 4.80-17.01). Travel costs were the main non-medical expenditure in all sites. Pre-treatment hospitalization costs were reported by 41 (16%) participants. After including the hospitalization cost, the median total cost was $35.45 (IQR 17.69-67.62) which was the highest for participants recruited in private hospitals ($54.51, IQR 29.48-98.47).

**Table 2:** Pre-treatment TB-related direct costs per person for participants with tuberculosis according to site of recruitment and main cost categories (N = 252).

Cost category	Community Health Center n = 81 (32%)		Private practitioner n = 58 (23%)		Public hospital n = 22 (9%)		Private hospital n = 91 (36%)	
	N (%)	Median (IQR)	N (%)	Median (IQR)	N (%)	Median (IQR)	N (%)	Median (IQR)
**Direct non-medical cost**	**77 (95)**	**6.85 (4.11-10.97)**	**22 (75)**	**8.88 (5.40-13.34)**	**22 (100)**	**7.71 (3.77-11.98)**	**58 (25)**	**9.33 (4.80-17.01)**
Travel	77 (95)	6.03 (4.01-9.60)	58 (100)	8.16 (5.12-13.20)	22 (100)	4.86 (2.65-10.28)	86 (95)	6.85 (4.11-11.79)
Food	28 (35)	1.37 (1.04-2.74)	21 (36)	3.08 (0.58-3.94)	9 (41)	3.42 (0.85-11.48)	45 (49)	4.11 (1.88-8.22)
Accommodation	1	0(0)	1	0 (0)	0	0(0)	7 (8)	6.85 (2.05-14.40)
**Direct medical cost**	**75 (93)**	**14.06 (5.28-29.56)**	**58 (100)**	**32.95 (14.02-52.10)**	**20 (91)**	**15.43 (8.40-36.35)**	**84 (92)**	**32.06 (9.73-74.83)**
Consultation	59 (73)	2.40 (0.82-6.85)	53 (91)	6.17 (1.92-9.53)	12 (55)	6.17 (2.74-13.02)	63 (69)	6.85 (2.74-23.31)
Sputum tests	18 (22)	8.22 (4.02-15.90)	21 (36)	7.54 (3.25-10.45)	7 (32)	4.11 (1.78-7.20)	37 (41)	8.91 (4.18-16.45)
Rapid Antigen test	3 (4)	7.89 (6.85)	4 (7)	15.42 (9.34-17.14)	0	0 (0)	17 (19)	6.78 (6.78-7.71)
PCR	0	0 (0)	0	0(0)	0	0(0)	7 (8)	33.94 (17.14-54.86)
Chest radiography	35 (43)	10.28 (5.48-17.14)	50 (86)	9.94 (3.68-20.31)	4 (18)	14.05 (9.61-21.17)	42 (46)	13.71 (9.17-19.03)
Medication^*^	69 (5)	6.85 (2.40-11.28)	55 (95)	10.28 (5.48-27.01)	18 (82)	10.62 (3.25-22.71)	78 (86)	13.81 (4.29-25.66)
**Sub-total cost per person** ^**^	**80 (99)**	**20.09 (10.42-36.32)**	**58 (100)**	**39.07 (21.79-66.50)**	**22 (100)**	**23.95 (12.85-44.74)**	**90 (99)**	**41.62 (17.46-80.47)**
Hospitalization	0	0(0)	0	0(0)	11 (50)	6.17 (2.74-14.40)	30 (33)	40.27 (19.99-96.50)
**Total direct cost** (including hospitalization)	**80 (99)**	**20.09 (10.42-36.32)**	**58 (100)**	**39.07 (21.79-66.50)**	**22 (100)**	**30.34 (15.61-30.34)**	**91 (100)**	**54.51 (29.48-98.47)**

Costs presented in the table above are in $US

US$=United States dollar; $1= 14,582 Indonesian Rupiah.

IQR=Interquartile range

* Medication could include antibiotics or treatment accessed from pharmacies or informal providers.

** Two participants (one from Community Health Centre and one from Private Hospital) spent nothing on pre-treatment cost (excluding hospitalization cost) and were not included in sub-total cost analysis.

### Cost proportions

**Fig 2:**
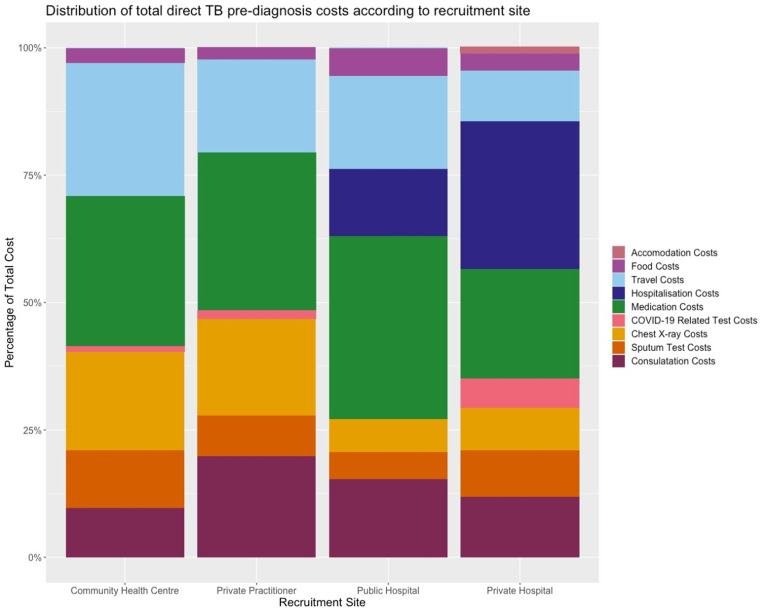
Distribution of total direct TB pre-diagnosis cost according to recruitment site.

Fig 2 shows pre-treatment medical costs related to medications was the highest proportion in participants recruited in CHCs (29%), public hospitals (36%), and private practitioners (31%), while in private hospitals, the highest proportion of costs was related to hospitalization (31%). The highest proportion of non-medical costs were for travel. COVID-19 screening (rapid antigen test and PCR) costs were absent from those recruited from public hospitals and were only a small proportion in participants recruited from the other sites.

### Factors associated with higher costs

Pre-treatment direct costs (excluding hospitalization) were significantly higher among those who had more than six visits (compared to less than six) to a healthcare provider before diagnosis (median $38.40 vs. $20.30; adjusted coefficient 18.10, p <  0.001), first visited a private hospital (median $50.68; adjusted coefficient 37.58, p =  0.001) or private practitioner (median $34.97; adjusted coefficient 18.17, p = 0.03) compared to a CHC, or were diagnosed in the private compared to public health sector (median $39.98 vs. $20.30; adjusted coefficient -13.71, p 0.002) (Table 3).

**Table 3:** Factors associated with median pre-treatment direct costs of participants with tuberculosis (N = 250).

Variable	Median Sub-total cost (IQR)	Unadjusted		Adjusted^*^	
**Coefficient (CI)**	**p-value**	**Coefficient (CI)**	**p-value**
Age					
18-39	27.98 (39.31)	–	–	–	**–**
40^+^	32.06 (40.85)	5.55 (-4.14-15.25)	0.26	-0.68 (-9.32-7.953)	0.88
Gender					
Male	29.15 (35.51)	–	–	–	**–**
Female	32.06 (46.55)	-3.84 (-13.21-5.53)	0.42	0.62 (-7.81-9.05)	0.88
Comorbidity					
No	28.80 (41.69)	–	–	–	–
Yes	33.40 (35.73)	4.59 (-5.85-15.04)	0.39	–	–
Education Level					
≥ High school completed	33.26 (46.63)	–	–	–	**–**
≤ Secondary completed	27.67 (32.32)	6.17 (-3.44-15.78)	0.21	–	–
Insurance Status					
Yes	27.47 (37.63)	–	–	–	**–**
No	37.60 (58.70)	-10.94 (-24.67-2.80)	0.12	-7.27 (-18.86-4.32)	0.22
Household members					
≤4	28.80 (39.91)	–	–	–	**–**
>4	30.86 (44.27)	2.06 (-7.41-11.53)	0.67	–	–
Income Earning Job					
No	26.67 (39.43)	–	–	–	**–**
Yes	33.26 (40.75)	-6.58 (-16.24-3.07)	0.18	-2.81 (-11.20-5.58)	0.51
Primary Income Earner					
Participant	30.65 (39.91)	–	–	–	**–**
Participant’s Family	28.80 (40.60)	-1.85 (-11.48-7.78)	0.70	–	–
Number of encounters with a health care provider before diagnosis					
<6	20.30 (32.16)	–	**–**	–	**–**
≥6	38.40 (47.52)	18.10 (8.07-28.14)	**<0.001**	18.10 (8.79-27.41)	**<0.001**
Duration from the first symptoms to diagnosis (days)					
<60	26.20 (33.81)	–	–	–	**–**
≥60	32.64 (47.66)	6.45 (-3.24-16.14)	0.19	-0.48(-9.63-8.67)	0.92
Provider at first encounter					
Community health Center	13.02 (16.72)	–	–	–	**–**
Informal provider	29.73 (39.16)	16.66 (0.31-33.02)	**0.046**	9.46 (-6.28-25.14)	0.24
Private practitioner	34.97 (48.0)	21.94 (4.98-38.91)	**0.011**	18.17 (1.94-34.40)	**0.03**
Private hospital	50.68 (65.27)	37.65 (15.68-59.61)	**<0.001**	37.58 (16.18-58.98)	**0.001**
Public hospital	18.89 (11.59)	11.66 (-39.68-62.99)	0.65	13.30 (-35.18-61.78)	0.59
Diagnosis site					
Public	20.30 (27.09)	–	–		
Private	39.98 (56.08)	-19.82 (-29.39-(-10.25))	**<0.001**	-13.71 (-22.48-(-4.95))	**0.002**

* Adjusted for age, gender, and other potential confounders

### Perspective and effect of the COVID-19 pandemic

In response to the question on the adequacy of household income to meet every day needs, most participants reported adequate income (85.7%) before the start of the COVID-19 pandemic but only 46% reported adequate income during the pandemic. 91 (36.1%) participants reported having some financial difficulties during the COVID-19 pandemic.

Participants reported they were still experiencing restrictions or quarantine protocols at the time of the interview (between July 2021 and February 2022). Some examples included mandatory mask wearing in public spaces (97.6%), social distancing (94.4%), restriction on large gatherings (73.0%), or full lockdown (42.1%). Moreover, there were also protocol changes in the usual health facility they visited, such as mandatory use of infection control measures (98.0%), screening for COVID-19 (66.3%), or reduced hours (26.0%). Subsequently, some participants reported that access to healthcare was affected. Almost half (44.8%) said that they had to wait longer than normal, 37.2% experienced complicated administrative requirements, and 12.7% were unable to reach a doctor because facilities were closed.

Being less willing to seek health care during the pandemic was reported by 153 (60.7%). This was mostly because of a fear of exposure to COVID-19 infection (43.7%), or not wanting to be tested or diagnosed with COVID-19 infection (28.2%). Just a small number of participants said that they could not afford to seek care because of lost income (3.2%).

### Coping mechanisms

Forty-eight participants reported borrowing money to cover costs due to their TB illness. This could be from different or a combination of sources but was mostly from family (n = 37; 77.0%), neighbors or friends (n = 12; 25.0%), or cooperative society (n = 1; 2.0%). Ten participants reported they sold their property, such as jewelry (80.0%), vehicle (20.0%), and/or other household items (10.0%).

## Discussion

In this study, we found that higher direct pre-treatment costs were associated with more than six visits to healthcare providers before diagnosis, first sought care at private hospital or private practitioner, or being diagnosed in the private health providers during the COVID-19 pandemic. Medication and diagnostic tests, especially chest radiography, were the major drivers for higher pre-treatment cost at almost all recruitment sites.

The median pre-treatment direct cost in this study was US$35.45. This cost was comparable with the previous study conducted in Bandung before COVID-19 pandemic (US$37.51) [10]. While in many countries, COVID-19-related diagnostic tests have been reported as leading to an increased out-of-pocket costs [27], in Indonesia, it is covered by the government. This policy is reflected in our study as the costs for COVID-19-related diagnostic tests are reported only by a few PWTB, especially those recruited at private practices or private hospitals. Although this could be seen as a good response from the health system in Bandung that they were able to continue the services for TB irrespective of the pandemic, there is a need to reduce either the pre-treatment costs or time to TB diagnosis. Furthermore, a previous study in Indonesia showed that a major cost incurred by patients was for personal protective equipment (PPE), such as masks, highlighting the need for more COVID-19-specific cost assistance measures [27].

Comparing pre-treatment costs data across countries, especially during the pandemic is difficult due to the data limitation. While the pre-COVID-19 studies showed a considerable range of cost for PWTB, such as Solomon Islands (US$6), Tamil Nadu, India (US$7.75), another part of Indonesia (US$16), and Bauchi State, Nigeria (US$28.99) [11,28–30] to the Ebonyi State, Nigeria (U$49) and a systematic review across nine studies in India (US$78.60)[31,32], this might partly due to different health systems.

In our study, PWTB who did not have health insurance seemed to have higher pre-treatment cost compare to those with insurance, although it was not statistically significant. PWTB who have health insurance can have less pre-treatment cost because TB related services are covered under the national insurance scheme plan in Indonesia [7,33], which include primary TB diagnosis through molecular testing (GeneXpert) and medications for drug-sensitive and drug-resistant TB. The goal of this coverage is to reduce the financial burden on PWTB and their households. This suggests that having insurance also becomes an enabler for patients to access health services, despite experiencing income loss.

Our study found that having an initial visit to a private hospital or private practitioner was significantly associated with higher costs, supporting results from another study in Indonesia that reported first visiting a private hospital resulted in significantly higher direct costs [11]. Moreover, a systematic review of studies conducted in India shared a similar finding of PWTB spending more money on pre-treatment or treatment if they first visited a clinician in the private health sector [32]. The private health providers appear to remain preferable as the first health care provider for initial care seeking and treatment by PWTB – often because of the convenience [11,32]. During the COVID-19 pandemic, this preference might have been increased because patients were fear of being infected by COVID-19 in the public healthcare services which that time were the major providers for COVID-19 related services [16].

Furthermore, our analysis also found that a risk factor for higher pre-diagnostic costs was having more than six visits to healthcare providers before diagnosis, similar to earlier study in Bandung [10]. A study in Ethiopia also showed a positive correlation with pre-treatment cost according to the number of healthcare facilities visited before diagnosis [34]. Having multiple visits to healthcare facilities not only leads to higher TB costs [35] but is also associated with diagnostic delays [5], which might aggravate the disease, increase risk of death and the possibility of the TB transmission in the community.

Our study is amongst a few to inquire about the out-of-pocket costs PWTB faced during the COVID-19 pandemic. However, our study does have some limitations. First, recruitment was only conducted in facilities that were willing to participate in the study, and the COVID-19 restrictions may have led to non-severe PWTB not seeking care. COVID-19 restrictions also limited the feasibility of recruiting PWTB’s carers, which led to the exclusion of PWTB with communicating problems altogether. Therefore, the study participants might not be representative of all PWTB in Bandung city during the COVID-19 pandemic. Second, the data that we collected from participants were self-reported and relied on participants’ recall thereby prone to recall bias. To minimize this recall bias, we recruited the study participants during the early phase of their treatment and some clinical information such as onset of symptoms, date of TB diagnosis, and TB treatment was validated by cross-checking medical records. Third, this study is COVID-19-period specific, meaning that the assessed effects of income losses in TB patients were also affected by COVID-19 situation. We have tried to assess patients’ situation as detailed as possible by maintaining adherence to WHO TBPCS methodology and adding COVID-19-related questions. However, we were still unable to disentangle the effects of TB independent of COVID-19 in the results. The study being COVID-19-period specific also relates to the challenges faced by research team to collect data amidst COVID-19 restrictions, which limited our capability to quantify postdiagnosis and catastrophic costs of TB. Lastly, as mentioned earlier, we did not interview participants who had successfully completed their TB treatment, so we could not quantify post-diagnosis and catastrophic costs.

## Conclusions

The overall pre-treatment costs experienced by PWTB during COVID-19 were still considerable and not dissimilar to pre-COVID-19, despite NTP providing free access to TB diagnosis diagnostic tests (GeneXpert) and treatment. However, the COVID-19 pandemic-related financial difficulties experienced – such as reduced household income - is likely to have impacted on these PWTB and their households. To reduce TB-related costs – and to protect PWTB from financial insecurity, it is imperative to strengthen the referral protocol among public and private health sectors, increase awareness regarding free TB diagnosis and treatment to the general population. Furthermore, insurance ownership, early detection of TB, and increasing the availability of TB diagnostics at private providers also play an important role in reducing the financial burden of pre-treatment cost.

## Supporting information

S1 Appendix Pre-treatment costs questionnaire. (PDF)
