# Supplementary material for: Pre-treatment direct costs for people with tuberculosis during the COVID-19 pandemic in different healthcare settings in Bandung, Indonesia
Source: PLoS One. 2025 Apr 1;20(4):e0320401. doi: 10.1371/journal.pone.0320401 (PMC11960889; doi:10.1371/journal.pone.0320401)
Supplement: S1 Appendix — (PDF) [file pone.0320401.s001.pdf]

Questionnaire health care pathways and out-of-pocket costs for patients diagnosed with TB or other respiratory diseases in Bandung amid COVID-19 pandemic

ID Subject :|_|_|_|_|_|_|_|
Subject initial :|_|_|_|_|

Form 1: Questionnaire healthcare pathways and out-of-pockets cost for patients diagnosed with TB or other respiratory diseases in Bandung amid COVID-19 pandemic

Notes

(green box) Not relevant for patients with respiratory diseases

Section 1: Healthcare pathways

No	Questions	Participant's responses
**1.**	Date of interview	|__|__|/|__|__|/|__|__|__|__| Day Month Year
**2.**	Interviewer initials	|__|__|__|
**3.**	Confirm whether the patient has agreed to participate?	|__| Yes<br>|__| No
**4.**	Confirm to TB officer, have patient been diagnosed with TB?	|__| Yes, patient diagnosed with TB (go to Q.**5**)<br>|__| No, patient diagnosed with other respiratory diseases (go to Q.**9**)
**5.**	Date of TB diagnosis (check AFB smear/GeneXpert result date)	|__|__|/|__|__|/|__|__|__|__| Day Month Year
**6.**	How was the diagnosis made?	|__| Positive AFB smear<br>|__| Clinical symptom and negative AFB smear<br>|__| Clinical symptom, negative AFB smear, and Positive Chest X-ray for TB<br>|__| Positive AFB smear and Positive Chest X-ray for TB<br>|__| Positive GeneXpert for MTB<br>|__| Other, specify: ________________________
**7.**	Which of these statements best describes the stage of TB care you are in right now?	|__| My doctor told me I have TB but I have not been given any medicines yet (Go to Q.9)<br>|__| I started taking TB medication, but I stopped before finishing all my medication (Go to Q.9)<br>|__| I am currently taking TB medication and have been taking my medicines up to today (Go to Q.8)<br>|__| I finished taking all my TB medication (Go to Q.9)<br>|__| Not sure (Go to Q.9)<br>|__| Other, specify: __________________________
**8.**	TB treatment period (in month)	|__| 1<br>|__| 2<br>|__| 3<br>|__| 4<br>|__| 5<br>|__| 6<br>|__| Other, specify_____ (fill with number)
**9.**	Recruitment site	|__| Private clinic, name: _________________<br>|__| Private practitioner (solo practice)<br>|__| Private specialist<br>|__| Private hospital, name: ___________________

Questionnaire health care pathways and out-of-pocket costs for patients diagnosed with TB or other respiratory diseases in Bandung amid COVID-19 pandemic

ID Subject :|_|_|_|_|_|_|_|
Subject initial :|_|_|_|_|

**Patient details**		
**10.**	Date of birth	|__|__|/|__|__|/|__|__|__|__| Day Month Year
**11.**	Age at time of interview	|__|__| Years
**12.**	Gender	|__| Male<br>|__| Female<br>|__| Other<br>|__| Prefer not to say
**13.**	Marital status	|__| Single<br>|__| Married<br>|__| Divorced<br>|__| Widowed<br>|__| Prefer not to say
**14.**	Have you had contact history with a person with TB?	|__| No<br>|__| Yes (specify: …………………..)<br>|__| Don't know
**15.**	Do you have any other illnesses?<br>*(check all that apply)*	1. HIV |__|Yes |__|No<br>2. Diabetes |__|Yes |__|No<br>3. Hypertension |__|Yes |__|No<br>4. Dyslipidemia (high cholesterol level) |__|Yes |__|No<br>5. Heart disease |__|Yes |__|No<br>6. Other (specify……………………) |__|Yes |__|No
**16.**	Within the last year, did you smoke?	|__|No |__|Yes
**Delay, pre-diagnosis and diagnosis costs**		
**17.**	a) What symptoms did you experience that led you to seek treatment for your <u>current TB illness</u>? *(Check all that apply)*<br><br>b) How long did you experience these symptoms <u>before</u> you went to seek treatment?	**a) <u>Symptoms</u>** **b) <u>Duration</u>**<br><br>|__| Cough ____days _____months<br>|__| Night sweats ____days _____months<br>|__| Coughing blood ____days _____months<br>|__| Weight loss ____days _____months<br>|__| Fever ____days _____months<br>|__| Chest congestion ____days _____months<br>|__| Head congestion ____days _____months<br>|__| Sore throat ____days _____months<br>|__| Rashes or allergies ____days_____months<br>|__|Trouble breathing/wheezing<br>____days _____months

		|__| Lack of appetite ____days _____months<br><br>|__| Other, specify:<br>1. ……………….. ____days _____months<br>2. ……………….. ____days _____months<br>3. ……………….. ____days _____months<br><br>|__| No symptoms (*specify why/how the patient was diagnosed with TB:……………………………*)
**18.**	Approximately what date did you <u>first</u> <u>start to experience the symptoms</u>?<br>*(If the patient cannot remember the exact date then just put the first day of the month and year)*	|__|__|/|__|__|/|__|__|__|__| Day Month Year
**19.**	Where did you seek treatment or advice for these symptoms?<br>*(Check all that apply and then the order of places where you went for treatment first, second, third etc.)*	**<u>Place</u>** **<u>Order</u>**<br>|__| Primary Health Care (name: ....) |__| |__| Tertiary hospital (RSHS) |__| |__| District hospital,<br>1. ………………………. |__| 2. ………………………. |__| 3. ………………………. |__| |__| Lung Hospital (RS Paru Rotinsulu) |__| |__| Lung clinic (BBKPM) |__| |__| Private hospital,<br>1. ………………………. |__| 2. ………………………. |__| 3. ………………………. |__| |__| Private practitioner (solo practice),<br>1. ………………………. |__| 2. ………………………. |__| 3. ………………………. |__| |__| Private specialist,<br>1. ………………………. |__| 2. ………………………. |__| 3. ………………………. |__| |__| Private Clinic<br>1. ………………………. |__| 2. ……………………….. |__| 3. ………………………. |__| |__| Pharmacy/ Drug store for medication |__| |__| Emergency room in a public facility |__| |__| Emergency room in a private facility |__| |__| Community health worker/CHEW |__| |__| Private Laboratory |__|

		|__| Traditional healer/ alternative medicine/ herbal therapy |__| |__| Other (specify ……………………) |__| |__| Not applicable (eg. because patient did not have any symptoms) |__|
**20.**	What date did you <u>first seek treatment</u> for your symptoms from a <u>formal</u> health care provider (ie. Puskesmas, Public, private hospital, private practitioner, specialist doctor) *(If the patient cannot remember the exact date then just put the first day of the month and year)*	|__|__|/|__|__|/|__|__|__|__| Day Month Year
**21.**	a) Why did you seek care at the <u>first formal health care provider</u> when you first realised that you were sick? *(i.e the <u>first formal</u> place mentioned in Q.19 – check all that apply*<br>b) Of the reasons you just mentioned, what were the <u>main reasons</u> for seeking care in that place? *(order the <u>main reasons</u> for seeking care in that place)*	<u>Reason</u> <u>Order</u><br>|__| Free/affordable |__| |__| Had used it/been there before |__| |__| Advised to go there |__| |__| Close to home |__| |__| Trust they can cure me |__| |__| Drugs are available |__| |__| Diagnostic services are available |__| |__| Better health care services |__| |__| Severe symptoms |__| |__| One-stop service: diagnostic and treatment services within the same place |__| |__| Other (specify ……………………) |__|
**22.**	Did you seek treatment at any stage from any <u>informal</u> place such as alternative medicine, herbal therapy or traditional healer?	|__| Yes, Approximate date<br>|__|__|/|__|__|/|__|__|__|__| Day Month Year<br>|__| No (go to Q.24)
**23.**	Why did you seek care at the <u>informal health care provider</u> when you first realised that you were sick?<br>*(Check all that apply)*	<u>Reason</u><br>|__| Free/affordable<br>|__| Had used it/been there before<br>|__| Advised to go there<br>|__| Close to home<br>|__| Trust they can cure me<br>|__| Drugs are available<br>|__| Better health care services<br>|__| Minor symptoms<br>|__| Other (specify ……………………..
**24.**	Please confirm, have you been diagnosed with TB?<br>(e.g. you have received test results that have said you are positive for TB)	|__| Yes (Go to Q.**25**)<br>|__| No (Go to Q.**39**)<br>|__| Not sure (Go to Q.**39**)

Questionnaire health care pathways and out-of-pocket costs for patients diagnosed with TB or other respiratory diseases in Bandung amid COVID-19 pandemic

ID Subject :|__|__|__|__|__|__|__|
Subject initial :|__|__|__|__|

**25.**	Did you receive antibiotics <u>before</u> your TB diagnosis?	1. |__| Yes, go to Q.26<br>2. |__| No, go to Q.27<br>3. |__| Can't remember/don't know, go to Q.27<br><br>If 'Yes', please write the name of antibiotics:<br>1. ……………………………………………………………<br>2. ……………………………………………………………<br>3. ……………………………………………………………
**26.**	How long were you treated with the antibiotics?	……………. (days)
**27.**	Where was your TB diagnosis <u>finally</u> made? *(<u>check only one</u>)*	|__| Primary Health Care (name…………………………)<br>|__| Public hospital (name ……………………)<br>|__| Tertiary hospital (RS Hasan Sadikin)<br>|__| Lung hospital (RS Paru Rotinsulu)<br>|__| Lung clinic (BBKPM)<br>|__| Private hospital (name…………………………)<br>|__| Private practitioner (name ……………………),<br>|__|Clinic<br>|__|Pharmacy<br>|__|Laboratory<br>|__| Private practitioner (solo practice)<br>|__| Private specialist (name………………………),<br>|__|Clinic<br>|__|Pharmacy<br>|__|Laboratory<br>|__| Private specialist (solo practice)<br>|__| Private Clinic (name………………………)<br>|__| Pharmacy/ Drug store for medication<br>|__| Emergency room in a public facility<br>|__| Emergency room in a private facility<br>|__| Community health worker/CHEW<br>|__| Private Laboratory<br>|__| Traditional healer/ alternative medicine/ herbal therapy<br>|__| Other (specify ……………………)
**28.**	Is the diagnostic site the same as the first location of seeking care?	|__| Yes, go to Q. 30<br>|__| No, go to Q. 29
**29.**	a) If the TB diagnosis site (Q.28) is different to where you first sought care (Q.19), why did you seek diagnosis at this clinic/hospital?<br>*(Check all that apply)*<br><br>b) Of the reasons you just mentioned what were the main reasons for diagnosis at	<u>Reason</u> — <u>Order</u><br>|__| Free/affordable — |__| |__| Referred by another health provider — |__| |__| Advised to go there by family/friends — |__| |__| Close to home — |__| |__| Used before — |__| |__| Not satisfied with other provider — |__| |__| It is a specialist hospital/clinic — |__| |__| Severe symptoms — |__| |__| Other (specify ………………………) — |__|

	this clinic *(order the main reasons for diagnosis at this site)*	
**30.**	Date started on TB treatment *(Check clinic records at recruitment site) (Note: if the exact day is not known then put the first day of the month)*	|__|__|/|__|__|/|__|__|__|__| Day Month Year
**31.**	Treatment category prescribed<br>*(Check clinic records at recruitment site)*	|__| Category I<br>|__| Category II<br>|__| Other (Specify …………………….)
**32.**	Where are you receiving your TB treatment from now?	|__| Primary Health Care (name..............................)<br>|__| Public hospital (name ……………………..)<br>|__| Tertiary hospital (RS Hasan Sadikin)<br>|__| Lung hospital (RS Paru Rotinsulu)<br>|__| Lung clinic (BBKPM)<br>|__| Private hospital (name.................................)<br>|__| Private practitioner (name ……………………..),<br>|__|Clinic<br>|__|Pharmacy<br>|__|Laboratory<br>|__| Private practitioner (solo practice)<br>|__| Private specialist (name.............................),<br>|__|Clinic<br>|__|Pharmacy<br>|__|Laboratory<br>|__| Private specialist (solo practice)<br>|__| Private Clinic (name............................)<br>|__| Pharmacy/ Drug store for medication<br>|__| Emergency room in a public facility<br>|__| Emergency room in a private facility<br>|__| Community health worker/CHEW<br>|__| Private Laboratory<br>|__| Traditional healer/ alternative medicine/ herbal therapy<br>|__| Other (specify ……………………..)
**33.**	Is the TB treatment site the same as the first location of TB diagnosis?	|__| Yes, go to Q. 35<br>|__| No, go to Q. 34
**34.**	a) If the TB treatment site (Q.32) is different to where you diagnosed with TB (Q.27), why did you get the treament at this clinic/hospital?	<u>Reason</u> <u>Order</u><br>|__| Free/affordable |__| |__| Referred by another health provider |__| |__| Advised to go there by family/friends |__| |__| Close to home |__| |__| Used before |__|

	(Check all that apply)<br>b) Of the reasons you just mentioned what were the main reasons for diagnosis at this clinic *(order the main reasons for diagnosis at this site)*	|__| Not satisfied with other provider |__| |__| It is a specialist hospital/clinic |__| |__| Severe symptoms |__| |__| Other (specify ....) |__|
**35.**	Please select the statement that best describes your accessibility to TB treatment<br><br>**(Read answer options)**	|__| I have <u>no challenges</u> accessing my TB medications, go to Q.37<br>|__| I have <u>some challenges</u> accessing my TB medications<br>|__| I have <u>many challenges</u> accessing my TB medications
**36.**	What challenges do you face when accessing your TB medications?<br><br>**(Read answer options and check all that apply)**	|__| Not being able to get my medicines on time/when I need them<br>|__| Being exposed to COVID-19<br>|__| People finding out I am on TB treatment<br>|__| Drug side effects<br>|__| High or unpredictable costs of treatment<br>|__| Transportation challenges<br>|__| Other, specify: ________________________
**37.**	What is the price of TB medication currently, per month?	Price: _________________________________ (put '0' if you were not charged)<br>|__| I am not on TB medications<br>|__| I do not know<br>|__| Prefer not to say
**38.**	How do your current healthcare costs in general compare to your costs before the COVID pandemic started in March 2020?<br><br>**Read answer options and check one**	|__| Costs decreased<br>|__| Costs stayed the same<br>|__| Costs increased<br>|__| Prefer not to say<br>|__| I do not know
**39.**	How were you instructed to collect your medication?<br>**(Read answer options)**	|__| Visit <u>THIS facility</u> to collect medication regularly<br>|__| Visit a <u>DIFFERENT clinical facility</u> to collect medication regularly<br>|__| Visit a pharmacy or drug shop to collect medication regularly<br>|__| Someone comes to your house to drop off the medication regularly<br>|__| Other, specify: _______
**40.**	How do your current healthcare costs in general compare to your costs before the COVID pandemic started in March 2020?	|__| Costs decreased<br>|__| Costs stayed the same<br>|__| Costs increased<br>|__| Prefer not to say<br>|__| I do not know

	**(Read answer options and check one)**	
**41.**	How long does it take to get from your home to the nearest Puskesmas?	____minutes _____hours
**42.**	What is the approximate distance from your home to the nearest Puskesmas?	|__||__| km<br>|__| Don't know/not sure
**43.**	What is the approximate distance from your home to the nearest private clinic, nursing home, or hospital?	|__||__| km<br>|__|Don't know/not sure
**44.**	How often do you use the internet for accessing healthcare? i.e. investigating symptoms, consultations on what medications to take, learning about prognosis of past diseases	|__| Daily<br>|__| More than once a week<br>|__| Once a week<br>|__| Less than once a week but more than once a month<br>|__| Once a month or less<br>|__| Never<br>|__| Other, specify: ________________________

Section 2: Out-of-pocket costs for TB patients

45. About how much did you (and your family/accompanying person) spend for each of the visits to an informal and formal health care provider* **before** you were diagnosed with TB or other respiratory disease, including the visit when you actually received your diagnosis? *Fill one line for each visit – even if there were several visits to the* ***same*** *provider. Make sure to include costs for the TB patient and their family/accompanying person if someone else went with them on any of these visits)*

**NB. A health care provider could include a registered clinic, traditional healer, pharmacy etc).*
Note: that even if a patient has to go to another puskesmas for sputum then this needs to be listed separately. Please do not include if a patient has been hospitalised

Visit	Provider	Approximate date	Time	Costs											
			Total time spent per visit (including travel time)	Administrative costs (registration, consultation)	Tests for sputum or other tests (excluding chest x-ray)	Test related to COVID-19			X-ray (including radiology fees and travel costs for x-ray) *List separately*	Drugs/ medicine	Travel (return total for visit)	Food	Accomm-odation	Pattern of care-seeking pathways:<br>**1= self-health care seeking**<br>**2= refer for diagnostic**<br>**3=refer for treatment**<br>**4= reverse referral**	Did you receive any insurance reimbursement?<br>yes:<br>no:
						rapid antibody	Antigen swab	PCR swab							
			___minutes<br>___hours												

	45b. What type of lab test(s) or diagnostic(s) did the provider recommend?								45c	45d
Did this **provider** tell you that you needed a lab test for your symptoms?<br><br>|__| Yes (go to Q. **45b)**<br>|__| No (go to next visit)<br>|__| Don't know/can't remember (go to next visit)	**Sputum test (microscopy)**<br><br>|__| Yes, with no difficulties<br>|__| Yes, with difficulties<br>|__| No, lab was closed<br>|__| No, I can't produce sputum<br>|__| No, I didn't have time<br>|__| Don't know/don't remember<br>|__|Other, specify:<br>_________	**Sputum test (GenXpert)**<br><br>|__| Yes, with no difficulties<br>|__| Yes, with difficulties<br>|__| No, lab was closed<br>|__| No, I can't produce sputum<br>|__| No, I didn't have time<br>|__| Don't know/don't remember<br>|__|Other, specify:<br>_________	**Antibody COVID test (blood)**<br><br>|__| Yes, with no difficulties<br>|__| Yes, with difficulties<br>|__| No, lab was closed<br>|__| No, I didn't have enough money<br>|__| No, I didn't have time<br>|__|No, the lab was too far<br>|__| Don't know/don't remember<br>|__|Other, specify:<br>_________	**Antigen COVID test (oral/nasal swab)**<br><br>|__| Yes, with no difficulties<br>|__| Yes, with difficulties<br>|__| No, lab was closed<br>|__| No, I didn't have enough money<br>|__| No, I didn't have time<br>|__|No, the lab was too far<br>|__| Don't know/don't remember<br>|__|Other, specify:<br>_________	**PCR COVID test (oral/nasal swab)**<br>|__| Yes, with no difficulties<br>|__| Yes, with difficulties<br>|__| No, lab was closed<br>|__| No, I didn't have enough money<br>|__| No, I didn't have time<br>|__|No, the lab was too far<br>|__| Don't know/don't remember<br>|__|Other, specify:<br>_________	**Chest X-ray**<br><br>|__| Yes, with no difficulties<br>|__| Yes, with difficulties<br>|__| No, lab was closed<br>|__| No, I didn't have enough money<br>|__| No, I didn't have time<br>|__|No, the lab was too far<br>|__| Don't know/don't remember<br>|__|Other, specify:<br>_________	**Blood test**<br><br>|__| Yes, with no difficulties<br>|__| Yes, with difficulties<br>|__| No, lab was closed<br>|__| No, I didn't have enough money<br>|__| No, I didn't have time<br>|__|No, the lab was too far<br>|__| Don't know/don't remember<br>|__|Other, specify:<br>_________	**Other, specify: ....**<br><br>|__| Yes, with no difficulties<br>|__| Yes, with difficulties<br>|__| No, lab was closed<br>|__| No, I didn't have enough money<br>|__| No, I didn't have time<br>|__|No, the lab was too far<br>|__| Don't know/don't remember<br>|__|Other, specify:<br>_________	In the case of a sputum test, where was sputum collected?<br><br>|__| At a private clinic/testing centre/ laboratory<br>|__| At a public facility/testing centre/laboratory<br>|__| At a mobile testing centre/camps<br>|__| Sputum collected at home<br>|__| Sputum test recommended, but patient did not follow through with the test.<br>|__| Other, specify: ______	How did the [**first provider**] communicate the sputum test results of the test with you?<br><br>|__| In person at the health facility<br>|__| They called me<br>|__| They sent me a text message<br>|__| They sent me an e-mail<br>|__| Other, specify:<br>__________

Visit	Provider	Approximate date	Time	Costs											
			Total <u>time</u> spent per visit (including travel time)	Administrative costs (registration, consultation)	Tests for sputum or other tests (excluding chest x-ray)	Test related to COVID-19			X-ray (including radiology fees and travel costs for x-ray) *List separately*	Drugs/ medicine	Travel (return total for visit)	Food	Accomm-odation	Pattern of care-seeking pathways:<br>**1= self-health care seeking**<br>**2= refer for diagnostic**<br>**3=refer for treatment**<br>**4= reverse referral**	Did you receive any insurance reimbursement?<br>yes:<br>no:
						rapid antibody	Antigen swab	PCR swab							
			___minutes<br>___hours												

Did this provider tell you that you needed a lab test for your symptoms?	45b. What type of lab test(s) or diagnostic(s) did the provider recommend?								45c	45d
	**Sputum test (microscopy)**	**Sputum test (GenXpert)**	**Antibody COVID test (blood)**	**Antigen COVID test (oral/nasal swab)**	**PCR COVID test (oral/nasal swab)**	**Chest X-ray**	**Blood test**	**Other, specify: ....**	In the case of a sputum test, where was sputum collected?	How did the [**first provider**] communicate the sputum test results of the test with you?
Did this **provider** tell you that you needed a lab test for your symptoms?<br><br>|__| Yes (go to Q. **45b)**<br>|__| No (go to next visit)<br>|__| Don't know/can't remember (go to next visit)	|__| Yes, with no difficulties<br>|__| Yes, with difficulties<br>|__| No, lab was closed<br>|__| No, I can't produce sputum<br>|__| No, I didn't have time<br>|__| Don't know/don't remember<br>|__|Other, specify: ________	|__| Yes, with no difficulties<br>|__| Yes, with difficulties<br>|__| No, lab was closed<br>|__| No, I can't produce sputum<br>|__| No, I didn't have time<br>|__| Don't know/don't remember<br>|__|Other, specify: ________	|__| Yes, with no difficulties<br>|__| Yes, with difficulties<br>|__| No, lab was closed<br>|__| No, I didn't have enough money<br>|__| No, I didn't have time<br>|__|No, the lab was too far<br>|__| Don't know/don't remember<br>|__|Other, specify: ________	|__| Yes, with no difficulties<br>|__| Yes, with difficulties<br>|__| No, lab was closed<br>|__| No, I didn't have enough money<br>|__| No, I didn't have time<br>|__|No, the lab was too far<br>|__| Don't know/don't remember<br>|__|Other, specify: ________	|__| Yes, with no difficulties<br>|__| Yes, with difficulties<br>|__| No, lab was closed<br>|__| No, I didn't have enough money<br>|__| No, I didn't have time<br>|__|No, the lab was too far<br>|__| Don't know/don't remember<br>|__|Other, specify: ________	|__| Yes, with no difficulties<br>|__| Yes, with difficulties<br>|__| No, lab was closed<br>|__| No, I didn't have enough money<br>|__| No, I didn't have time<br>|__|No, the lab was too far<br>|__| Don't know/don't remember<br>|__|Other, specify: ________	|__| Yes, with no difficulties<br>|__| Yes, with difficulties<br>|__| No, lab was closed<br>|__| No, I didn't have enough money<br>|__| No, I didn't have time<br>|__|No, the lab was too far<br>|__| Don't know/don't remember<br>|__|Other, specify: ________	|__| Yes, with no difficulties<br>|__| Yes, with difficulties<br>|__| No, lab was closed<br>|__| No, I didn't have enough money<br>|__| No, I didn't have time<br>|__|No, the lab was too far<br>|__| Don't know/don't remember<br>|__|Other, specify: ________	|__| At a private clinic/testing centre/ laboratory<br>|__| At a public facility/testing centre/laboratory<br>|__| At a mobile testing centre/camps<br>|__| Sputum collected at home<br>|__| Sputum test recommended, but patient did not follow through with the test.<br>|__| Other, specify: ______	|__| In person at the health facility<br>|__| They called me<br>|__| They sent me a text message<br>|__| They sent me an e-mail<br>|__| Other, specify: __________

<table>
<tr><td rowspan="3">Visit</td><td rowspan="3">Provider</td><td rowspan="3">Approximate date</td><td>Time</td><td colspan="12">Costs</td></tr>
<tr><td rowspan="2">Total <u>time</u> spent per visit (including travel time)</td><td rowspan="2">Administrative costs (registration, consultation)</td><td rowspan="2">Tests for sputum or other tests (excluding chest x-ray)</td><td colspan="3">Test related to COVID-19</td><td rowspan="2">X-ray (including radiology fees and travel costs for x-ray) List separately</td><td rowspan="2">Drugs/ medicine</td><td rowspan="2">Travel (return total for visit)</td><td rowspan="2">Food</td><td rowspan="2">Accomm-odation</td><td rowspan="2">Pattern of care-seeking pathways:
1= self-health care seeking
2= refer for diagnostic
3=refer for treatment
4= reverse referral</td><td rowspan="2">Did you receive any insurance reimbursement?
yes:
no:</td></tr>
<tr><td>rapid antibody</td><td>Antigen swab</td><td>PCR swab</td></tr>
<tr><td rowspan="3"></td><td rowspan="3"></td><td rowspan="3"></td><td>___minutes
___hours</td><td></td><td></td><td></td><td></td><td></td><td></td><td></td><td></td><td></td><td></td><td></td><td></td></tr>
</table>

<table>
<tr><td rowspan="2">Did this provider tell you that you needed a lab test for your symptoms?

|__| Yes (go to Q. 45b)
|__| No (go to next visit)
|__| Don't know/can't remember (go to next visit)</td><td colspan="8">45b. What type of lab test(s) or diagnostic(s) did the provider recommend?</td><td>45c</td><td>45d</td></tr>
<tr><td>Sputum test (microscopy)

|__| Yes, with no difficulties
|__| Yes, with difficulties
|__| No, lab was closed
|__| No, I can't produce sputum
|__| No, I didn't have time
|__| Don't know/don't remember
|__|Other, specify:
_________</td><td>Sputum test (GenXpert)

|__| Yes, with no difficulties
|__| Yes, with difficulties
|__| No, lab was closed
|__| No, I can't produce sputum
|__| No, I didn't have time
|__| Don't know/don't remember
|__|Other, specify:
_________</td><td>Antibody COVID test (blood)

|__| Yes, with no difficulties
|__| Yes, with difficulties
|__| No, lab was closed
|__| No, I didn't have enough money
|__| No, I didn't have time
|__|No, the lab was too far
|__| Don't know/don't remember
|__|Other, specify:
_________</td><td>Antigen COVID test (oral/nasal swab)

|__| Yes, with no difficulties
|__| Yes, with difficulties
|__| No, lab was closed
|__| No, I didn't have enough money
|__| No, I didn't have time
|__|No, the lab was too far
|__| Don't know/don't remember
|__|Other, specify:
_________</td><td>PCR COVID test (oral/nasal swab)
|__| Yes, with no difficulties
|__| Yes, with difficulties
|__| No, lab was closed
|__| No, I didn't have enough money
|__| No, I didn't have time
|__|No, the lab was too far
|__| Don't know/don't remember
|__|Other, specify:
_________</td><td>Chest X-ray

|__| Yes, with no difficulties
|__| Yes, with difficulties
|__| No, lab was closed
|__| No, I didn't have enough money
|__| No, I didn't have time
|__|No, the lab was too far
|__| Don't know/don't remember
|__|Other, specify:
_________</td><td>Blood test

|__| Yes, with no difficulties
|__| Yes, with difficulties
|__| No, lab was closed
|__| No, I didn't have enough money
|__| No, I didn't have time
|__|No, the lab was too far
|__| Don't know/don't remember
|__|Other, specify:
_________</td><td>Other, specify:
....
|__| Yes, with no difficulties
|__| Yes, with difficulties
|__| No, lab was closed
|__| No, I didn't have enough money
|__| No, I didn't have time
|__|No, the lab was too far
|__| Don't know/don't remember
|__|Other, specify:
_________</td><td>In the case of a sputum test, where was sputum collected?

|__| At a private clinic/testing centre/ laboratory
|__| At a public facility/testing centre/laboratory
|__| At a mobile testing centre/camps
|__| Sputum collected at home
|__| Sputum test recommended, but patient did not follow through with the test.
|__| Other, specify: ______</td><td>How did the [first provider] communicate the sputum test results of the test with you?

|__| In person at the health facility
|__| They called me
|__| They sent me a text message
|__| They sent me an e-mail
|__| Other, specify:
__________</td></tr>
</table>

Nomor ID Studi :|_|_|_|_|_|_|_|_|
Inisial Subjek :|_|_|_|_|_|

**Insurance**		
**46.**	What type of insurance do you have? *(check all that apply)*	|__| BPJS (Government insurance)<br>|__| Private insurance (specify: ....)<br>|__| No insurance<br>|__| Other (specify.................................)
**47.**	Have you received any reimbursement for any costs related to your TB illness?	|__| Yes (go to Q.**48)**<br>|__| No (go to Q.**49**)<br>|__| Don't know (go to Q.**49**)<br>|__| Patient refuse to answer (go to Q.**49**)
**48.**	How much have you received?	Total: Rp ....<br>|__| Patient refuse to answer
**Coping costs**		
**49.**	Did you borrow any money to cover costs due to your TB illness?	|__| Yes<br>|__| No (go to Q.**52**)<br>|__| Patient refuse to answer (go to Q.**52**)
**50.**	How much did you borrow?	Total: Rp ....<br>|__| Patient refuse to answer
**51.**	From whom did you borrow this money? *(Check all that apply)*	|__| Family<br>|__| Neighbours/friends<br>|__| Private/public loan provider<br>|__| Pegadaian<br>|__| Private/public bank<br>|__| Cooperative<br>|__| Other (specify......................................)
**52.**	Have you sold any of your property to finance the cost of your TB illness?	|__| Yes<br>|__| No (go to Q.**55**)<br>|__| Patient refuse to answer (go to Q.**55**)
**53.**	What have you sold? *(check all that apply)*	|__| Land<br>|__| House<br>|__| Livestock<br>|__| Transport/vehicle<br>|__| Jewellery<br>|__| Household item<br>|__| Other (specify......................................)
**54.**	How much did you earn from the sale of all of the above items mentioned?	Total: Rp ....<br>|__| Patient refuse to answer
**Socioeconomic information (individual situation and income)**		
**55.**	Who is the <u>primary</u> income earner in the household? *(check only one)*	|__| TB patient<br>|__| Wife<br>|__| Husband

Nomor ID Studi :|_|_|_|_|_|_|_|_|
Inisial Subjek :|_|_|_|_|

		|__| Parent<br>|__| Son/daughter<br>|__| Other extended family<br>|__| Other (specify…………………………)
**56.**	What type of dwelling do you currently live in?	|__| House/apartment/flat<br>|__| Rented house/boarding house<br>|__| Informal housing<br>|__| Other, specify: ___________________
**57.**	Do you (or your spouse/partner) own or rent your dwelling?	|__| Own outright<br>|__| Own on mortgage<br>|__| Rent from local authority/housing association<br>|__| Rent from private landlord<br>|__| Other, specify: ___________________
**58.**	How many individuals (relationship, age, and gender) live with you?<br><br>**For each person that lives with you specify their relationship to you, their age, and their gender:**	|__||__| people

Relationship	Age (years)	Gender (M/F)

**59.**	What is <u>your</u> highest level of education? *(check only one)*	|__| No formal schooling<br>|__| Less than primary school<br>|__| Primary school completed<br>|__| Secondary school completed<br>|__| High school completed<br>|__| College completed<br>|__| University completed<br>|__| Post-graduate completed: magister<br>|__| Post-graduate completed: doctoral<br>|__| Other, specify: ____________________
**60.**	What is your current employment status?	|__| Working<br>(go to Q.**61**)<br>|__| Self-employed

Nomor ID Studi :|_|_|_|_|_|_|_|_|
Inisial Subjek :|_|_|_|_|

		(go to Q.**62**)<br>|__| Unemployed/underemployed (go to Q.**64**)<br>|__|Retired (go to Q.**64**)<br>|__| Lain-lain (sebutkan ....) (go to Q.**61**)
**61.**	What is your main occupation?	|__| Civil servant/Indonesian Army/Police<br>|__| Private employee<br>|__| Factory workers<br>|__| Teacher<br>|__| Seller<br>|__| Other, specify: ____________________
**62.**	Are you currently working?	|__| Yes (go to Q.**63**)<br>|__| No (go to Q.**64**)
**63.**	Which of the following describes your working schedule?<br><br>(go to Q.66)	|__| Daytime shift<br>|__| Evening shift<br>|__| Night shift<br>|__| Rotating shift<br>|__| Seasonal, on-call, no pre-arranged schedules<br>|__| Other, specify: ____________________
**64.**	If not working, how long have you been unemployed?	|__| Unemployed as of this month<br>|__| 1-2 months<br>|__| 2-6 months<br>|__| Over 6 months<br>|__| Never been employed
**65.**	What would best describe the reason for not working?	|__| Unable to work because of sickness or disability<br>|__| Looking after family<br>|__| Student<br>|__| Doing unpaid work<br>|__| Laid off due to COVID-19 pandemic<br>|__| Laid off due to some other reason than COVID-19 pandemic<br>|__| Other, specify: ____________________
**66.**	Is the reason for not working related to your TB illness?	|__| Yes<br>|__| No
**67.**	What was your estimated <u>personal</u> take home earning per month BEFORE the TB illness?	.... Total IDR<br>|__| Don't earn<br>|__|Patient refused to answer
**68.**	What is your estimated <u>personal</u> take home earning per month NOW?	.... Total IDR<br>|__| Don't earn<br>|__|Patient refused to answer

Nomor ID Studi :|_|_|_|_|_|_|_|_|
Inisial Subjek :|_|_|_|_|_|

**69.**	Are there any changes in your income after you were diagnosed with TB?	|__| Yes<br>|__| No (go to Q.**73**)
**70.**	If answer to Q.68 differs to Q.67<br>Do you think the change in earnings related to your TB illness?	|__| Yes<br>|__| No
**71.**	Have you ever stopped working/going to school/university/doing housework because of your TB?	|__| Yes<br>|__| No (go to Q.**73**)
**72.**	For how long?	|__| Less than 1 month<br>|__| 1 month<br>|__| 2-3 months<br>|__| 4-5 months<br>|__| More than 6 months
**73.**	How would you describe your financial standing **before the start of the COVID-19 Pandemic in March 2020**?<br><br>**Read answer options**	|__| Adequate (my family and I can meet our needs every month)<br>|__| Barely adequate (e.g., some months my family and I can meet our needs, others we cannot)<br>|__| Inadequate (e.g., most months my family and I cannot meet our needs)
**74.**	Which of these phrases best describes how you (and your spouse/partner) are getting along financially now (during the COVID-19 pandemic)?	|__| Manage very well<br>|__| Manage quite well<br>|__| Get by alright<br>|__| Don't manage very well<br>|__| Have some financial difficulties<br>|__| Have severe financial difficulties
**75.**	How would you describe your **current** financial standing?	|__| Adequate<br>|__| Barely adequate<br>|__| Inadequate
**76.**	Have you had any other household expenses not already mentioned related to the time you have been unwell with TB (for example, having to employ someone else to do your work or household work)	|__| Yes (specify ....)<br>|__| No (go to Q.78)
**77.**	How much have you paid in total for these extra costs?	.... total IDR
**78.**	Do you have children of or below school age?	|__| Yes (how many?............)<br>|__| No (go to Q.82)
**79.**	Do all of your children of school age attend school regularly?	|__| Yes (go to Q.82)<br>|__| No (go to Q.80)
**80.**	Why not?	|__| Need to help in the house<br>|__| No money for school fees<br>|__| Needs to work to earn money<br>|__| Also sick<br>|__| Other (specify ....)
**81.**	Do any of your children of or below school age, work or are not able to attend school due to your TB illness?	|__| Yes<br>|__| No

**82.**	Has your TB illness affected your social or private life in any way?<br>(*Check all that apply)*	|__| No effect/problems<br>|__| Separated from spouse<br>|__| Discriminated at work<br>|__| Stopped socialising with family and friends<br>|__| Affected self-esteem<br>|__| Other (specify ....)
**83.**	How many of your household members are <u>normally</u> paid for working? *(including the TB patient if in paid work before the TB illness)*	....
**84.**	How much do you estimate was the average income of your <u>household</u> per month BEFORE the TB illness? *(all the income from all household members in paid work*)	.... total IDR<br>|__| don't know<br>|__| Patient refused to answer
**85.**	How much do you estimate is the average income of your <u>household</u> per month NOW? *(all the income from all household members in paid work*)	.... total IDR<br>|__| don't know<br>|__| Patient refused to answer
**86.**	How many people regularly sleep in your house? (including you)	....
**87.**	Besides you, is anyone else in your household on treatment for TB?	|__| Yes (how many people...................)<br>|__| No

Bagian 3: COVID-19 Pandemic

**Perspectives on the COVID-19 pandemic**		
**88.**	At any point between March 2020 and now, have there been any COVID-19 lockdowns or restrictions in place around where you live/your neighbourhood?	|__| Yes<br>|__| No<br>|__| Don't know
**89.**	Which of the following COVID-19 related restrictions or protocols **are currently in place** you live/in your neighbourhood?<br><br>**Read answer options and check all that apply**	|__| Movement completely restricted, full lockdown (e.g., all stores, restaurants, bars, churches, and mosques closed)<br>|__| Restaurants and bars are open with limited capacity<br>|__| Markets and stores open with limited capacity<br>|__| Religious ceremonies and services at churches and mosques at limited capacity<br>|__| Movement partially restricted (e.g., curfew, daily walks/exercise permitted)<br>|__| Masks mandatory in public spaces<br>|__| Social distancing mandated<br>|__| Large gatherings are restricted<br>|__| Other, (specify ....)
**90.**	Which of the following COVID-19 related restrictions or protocols	|__| Movement completely restricted, full lockdown (e.g., all stores, restaurants, bars, churches, and mosques closed)

	**have ever been in place** you live/in your neighbourhood?<br><br>**Read answer options and check all that apply**	|__| Restaurants and bars are open with limited capacity<br>|__| Markets and stores open with limited capacity<br>|__| Religious ceremonies and services at churches and mosques at limited capacity<br>|__| Movement partially restricted (e.g., curfew, daily walks/exercise permitted)<br>|__| Masks mandatory in public spaces<br>|__| Social distancing mandated<br>|__| Large gatherings are restricted<br>|__| Other, (specify ……………………..)
**91.**	Which statement best describes your overall experience accessing healthcare during the COVID-19 pandemic (compared your access before the pandemic)?<br><br>**Read answer options and check all that apply**	|__| It is easier to access healthcare<br>|__| It is more difficult to access healthcare<br>|__| Access to healthcare stayed the same
**92.**	Select the statement(s) that reflect how COVID-related lockdowns or restrictions have affected your access to healthcare since the pandemic started in March 2020.<br><br>**Read answer options and check all that apply**	|__| I was unable to reach a doctor because facilities were closed due to the COVID-19 pandemic<br>|__| I was unable to receive my medications because drug stores were closed due to the COVID-19 pandemic<br>|__| I had to seek care from a different facility than my typical/preferred facility<br>|__| Waiting times at facilities were longer than normal/expected<br>|__| I was unable to leave the house to seek care because of movement/transport restrictions<br>|__| Other, (specify ……………………..)
**93.**	How frequently do you go outside your house/apartment/flat?	|__| Daily<br>|__| Less than daily but more than once a week<br>|__| Once a week<br>|__| Less than once a week
**94.**	Which of the following are the reason(s) you go outside of your house/apartment/flat?<br><br>**Read answer options and check all that apply**	|__| Go to the market or other errands<br>|__| Seek healthcare or medicine<br>|__| Drop off/pick-up children from school/nursery<br>|__| Work<br>|__| School<br>|__| Exercising<br>|__| Socializing with family, neighbours, or friends<br>|__| Attend religious gathering at a church, mosque, etc.<br>|__| Other, (specify ……………………..)
**95.**	Have **you or any of your close friends and family** been diagnosed with COVID-19?	|__| Yes<br>|__| No (go to Q.97)

Nomor ID Studi :|_|_|_|_|_|_|_|_|
Inisial Subjek :|_|_|_|_|

**96.**	Choose the statement(s) which characterises **your personal or immediate family experience** with COVID-19.<br><br>**Read answer options and check all that apply**	|__| Diagnosed with COVID-19 but experienced mild/moderate symptoms<br>|__| Very sick with COVID-19 for a short period (Two weeks or less)<br>|__| Very sick with COVID 19 for a long period (More than two weeks)<br>|__| Hospitalized with COVID 19<br>|__| Death due to COVID 19 (in case of a loved one)<br>|__| Other, specify
**97.**	Has COVID and/or current/past COVID-related restrictions affected *your willingness* to seek healthcare?	|__| Yes, I am more willing to seek care (go to Q.99)<br>|__| Yes, I am less willing to seek care (go to Q.98)<br>|__| No effect (go to Q.100)<br>|__| Don't know/not sure (go to Q.100)
**98.**	Select the statement(s) that describe how COVID has *decreased* your willingness to seek health care.<br><br>**Read answer options and check all that apply**	|__| I am afraid of visiting health facilities because of a fear of exposure to COVID-19 infection<br>|__| I am afraid of visiting health facilities because I don't want to be tested or diagnosed with COVID-19 infection<br>|__| I am less able to/can no longer afford to seek care because of lost income in the past year<br>|__| Other, (specify ……………………….)
**99.**	Select the statement(s) that describe how COVID has *increased* your willingness to seek health care.<br><br>**Read answer options and check all that apply**	|__| I am more aware that early healthcare seeking is important<br>|__| I have to get tested regularly for work/school<br>|__| I am worried about infecting my family and friends therefore I seek healthcare more often<br>|__| I am worried about getting infected from people I come in contact with therefore I seek healthcare more often<br>|__| I want to see my family and therefore as a precaution I get tested and seek extra care<br>|__| My underlying/chronic condition makes me more willing to seek care during the COVID-19 pandemic<br>|__| Other, specify
**Health Care Utilization during the COVID-19 pandemic**		
**100.**	In general, how would you describe your overall health (including mental health)?<br><br>**Read answer options**	|__| Excellent<br>|__| Very good<br>|__| Good<br>|__| Fair<br>|__| Poor
**101.**	Compared with before March 01, 2020, how would you describe your health now?<br><br>**Read answer options**	|__| Better<br>|__| Worse<br>|__| About the same
**102.**	Including this facility, what are all the different types of health	|__| Primary Health Care (name................................)<br>|__| Public hospital (name ....)

<table>
<tr><td></td><td>providers you have had contact with about your physical or mental health since the beginning of the COVID-19 pandemic?

Read answer options and check all that apply.

Remind patient they should count the current facility, in addition to any others consulted since March 2020</td><td>|__| Tertiary hospital (RS Hasan Sadikin)
|__| Lung hospital (RS Paru Rotinsulu)
|__| Lung clinic (BBKPM)
|__| Private hospital (name................................)
|__| Private practitioner (name ....),
|__|Clinic
|__|Pharmacy
|__|Laboratory
|__| Private practitioner (solo practice)
|__| Private specialist (name.............................),
|__|Clinic
|__|Pharmacy
|__|Laboratory
|__| Private specialist (solo practice)
|__| Private Clinic (name.............................)
|__| Pharmacy/ Drug store for medication
|__| Emergency room in a public facility
|__| Emergency room in a private facility
|__| Community health worker/CHEW
|__| Private Laboratory
|__| Traditional healer/ alternative medicine/ herbal therapy
|__| Other (specify ……………………..)</td></tr>
<tr><td>103.</td><td>When you get sick and you decide to seek treatment, where do you typically go to seek help <u>first</u>?

Read answer options</td><td>|__| Primary Health Care (name................................)
|__| Public hospital (name ....)
|__| Tertiary hospital (RS Hasan Sadikin)
|__| Lung hospital (RS Paru Rotinsulu)
|__| Lung clinic (BBKPM)
|__| Private hospital (name................................)
|__| Private practitioner (name ....),
|__|Clinic
|__|Pharmacy
|__|Laboratory
|__| Private practitioner (solo practice)
|__| Private specialist (name.............................),
|__|Clinic
|__|Pharmacy
|__|Laboratory
|__| Private specialist (solo practice)
|__| Private Clinic (name.............................)
|__| Pharmacy/ Drug store for medication
|__| Emergency room in a public facility
|__| Emergency room in a private facility
|__| Community health worker/CHEW
|__| Private Laboratory
|__| Traditional healer/ alternative medicine/ herbal therapy
|__| Other (specify ……………………..)</td></tr>
</table>

**104.**	How has the health facility you go to most frequently been affected by the COVID-19 pandemic?<br><br>**Read answer options and check all that apply**	|__| Closed<br>|__| Reduced hours<br>|__| Reduced services<br>|__| Screening for COVID-19<br>|__| Increased cost<br>|__| Decreased cost<br>|__| Infection control measures (spacing (as in physicial distancing), mandatory mask-wearing, hand washing, hand sanitizing stations, etc.)<br>|__| No change/has not been affected<br>|__| Other, (specify ……………………..)
**105.**	Since March 01, 2020, was there ever a time when you felt that you needed health care but didn't receive it?	|__| Yes<br>|__| No (go to Q.**107**)<br>|__| Can't remember (go to Q.**107**)
**106.**	Thinking of the most recent time, why did you not receive it?<br><br>**Read answer options and check all that apply**	|__| Healthcare was not available because practice was closed due to the COVID-19 pandemic<br>|__| Healthcare was not available because practice was closed, but not due to COVID-19<br>|__| Waiting time was too long<br>|__| Could not afford<br>|__| Too busy with work and other responsibilities<br>|__| Decided not to seek care due to fear of exposure to COVID-19 pandemic<br>|__| Other, (specify ……………………..)
**107.**	Since the start of the COVID pandemic in March 2020, have you had any problems obtaining any non-TB-related medications you needed?	|__| Yes<br>|__| No (go to Q.**109**)<br>|__| Can't remember (go to Q.**109**)
**108.**	Which of the following best describe the reasons you have had trouble obtaining any non-TB-related medicines?<br><br>**Read answer options and check all that apply**	|__| Pharmacy/provider was closed<br>|__| Pharmacy/provider was understocked<br>|__| I had problems getting to the pharmacy<br>|__| I could not afford the medicine<br>|__| I don't have health insurance<br>|__| Other problem, (specify ……………………..)
**109.**	We would like to know how frequently you have used telemedicine. Telemedicine includes using phone calls, video calls, WhatsApp, or online "virtual visits" to consult with a health care provider.<br><br>**Before the start of the COVID-pandemic**, how frequently were	|__| Did not use telemedicine<br>|__| Daily<br>|__| More than once a week<br>|__| Once a week<br>|__| Less than once a week but more than once a month<br>|__| Once a month or less<br>|__| Other, (specify ……………………..)

	you using telemedicine to consult with a health care professional?	
**110.**	<u>**Since the start of COVID-pandemic**</u> in March 2020, how frequently have you been using telemedicine to consult with a health care professional?	|__| Did not use telemedicine<br>|__| Daily<br>|__| More than once a week<br>|__| Once a week<br>|__| Less than once a week but more than once a month<br>|__| Once a month or less<br>|__| Other, (specify ……………………..)
